# An Unsupervised Deep Feature Learning Model Based on Parallel Convolutional Autoencoder for Intelligent Fault Diagnosis of Main Reducer

**DOI:** 10.1155/2021/8922656

**Published:** 2021-09-30

**Authors:** Qing Ye, Changhua Liu

**Affiliations:** ^1^School of Computer Science, Yangtze University, Jingzhou 430023, China; ^2^General Office, Yangtze University, Jingzhou 430023, China

## Abstract

Traditional diagnostic framework consists of three parts: data acquisition, feature generation, and fault classification. However, manual feature extraction utilized signal processing technologies heavily depending on subjectivity and prior knowledge which affect the effectiveness and efficiency. To tackle these problems, an unsupervised deep feature learning model based on parallel convolutional autoencoder (PCAE) is proposed and applied in the stage of feature generation of diagnostic framework. Firstly, raw vibration signals are normalized and segmented into sample set by sliding window. Secondly, deep features are, respectively, extracted from reshaped form of raw sample set and spectrogram in time-frequency domain by two parallel unsupervised feature learning branches based on convolutional autoencoder (CAE). During the training process, dropout regularization and batch normalization are utilized to prevent over fitting. Finally, extracted representative features are feed into the classification model based on deep structure of neural network (DNN) with softmax. The effectiveness of the proposed approach is evaluated in fault diagnosis of automobile main reducer. The results produced in contrastive analysis demonstrate that the diagnostic framework based on parallel unsupervised feature learning and deep structure of classification can effectively enhance the robustness and enhance the identification accuracy of operation conditions by nearly 8%.

## 1. Introduction

As the key and principal component of the automobile transmission system, rear axle is of great significance to driving security and comfort of automobile. According to the previous investigation and experience of experts, the faults of the automobile rear axle mainly occur in the main reducer which is the core component of rear axle [1–3]. Therefore, condition monitoring and fault identification of main reducer can effectively identify various failure types and implement maintenances in time to avoid catastrophic accidents [4].

Machine health monitoring can be achieved by analyzing vibration signals, current signals, acoustical signals, and temperature data. In recent years, signal processing technique based on vibration signals is the most commonly used and effective approach for traditional condition monitoring and failure recognition [5–7]. For rotating machinery, vibration signals collected from accelerometer have characteristics of nonlinear, nonstationary, high dimensional, and transient which obviously increase the difficulty of fault identification [8].

In traditional fault diagnosis, signal processing techniques can be implemented in time domain, frequency domain, and time-frequency domain for feature extraction before failure recognition [9, 10]. Features embedded in vibration signals can reflect the trend of status variance, and features in frequency domain can compress noise to separate pure signal from vibration signal with strong noise while dimensionality of these features is still as high as original signals [11]. Time-frequency analysis approaches such as empirical mode decomposition (EMD) [12] and continuous wavelet transform (CWT) [7] are superior to time-domain analysis and frequency-domain analysis in extracting valuable details of signals by telescopic translation technique [13–15]. Feature extraction based on signal processing technologies is not general for all kinds of signals and heavily depends on manual selection of features and prior knowledge during the process of analysis, which seriously affects the performance [16, 17].

By introducing some machine learning algorithms and deep learning models into the mechanical field, condition monitoring and fault identification of mechanical equipment based on support vector machine (SVM) [18], artificial neural networks (ANNs) [19], stacked autoencoder (SAE) [20, 21], convolutional neural networks (CNNs) [22, 23], and deep neural networks (DNNs) [24, 25] have gradually become a research hotspot [26]. In practical applications, a large number of dataset with labels are generally hard to collect due to time cost and labor consumption. Accordingly, among the state-of-the-art deep learning techniques, the unsupervised deep feature learning model based on SAE and other variants have shown an important role in some existing applications of machinery condition monitoring and fault diagnosis [27]. Godói utilized some variant of SAE in condition monitoring of rotating machines [28]. Huan Chen et al. proposed a convolutional autoencoder-based method for energy disaggregation [29]. Aljemely et al. adopted deep functional autoencoder to generate a deep feature extraction approach for failure recognition [30].

Furthermore, deep belief network (DBN) is another typical unsupervised learning method which is widely used in feature extraction and dimensional reduction [31]. Peng et al. utilized multilayer DBN to extract deep features of engine sensing data [32]. Deutsch et al. adopted DBN and particle filtering method to mine the degraded feature information [33]. Hu et al. proposed a high-dimensional fault feature extraction method based on DBN [34]. As similar as SAE, DBN is also trained using the unsupervised greedy pretraining method layer-by-layer. However, since DBN has poor ability to learn features from sequential signal, it subjects certain restrictions in processing sequential vibration data for intelligent fault diagnosis of machinery.

With the purpose of handling the shortcomings of various feature extraction methods based on traditional signal processing and further improving the veracity of fault identification, a novel unsupervised deep feature learning approach based on parallel convolutional autoencoder (PCAE) is proposed, which combines with DNN together to achieve operation condition monitoring and failure identification of main reducer. The contributions of this research are summed up into the following three points:

1. To utilize respective advantages of various modal features, in this research, we facilitate information fusion in feature extraction. Deep features are automatically extracted from segmentations of raw signals and spectrogram in time-frequency domain by two parallel feature learning branches based on convolutional autoencoder.
2. During the training process of unsupervised deep feature extraction, we utilize dropout regularization technique to prevent overfitting. Additionally, batch normalization is utilized to sensibly adjust the output value of each layer in the deep structure so as to relieve gradient vanishing and improve training speed.
3. In this research, we present an intelligent failure identification framework for main reducer based on PCAE and DNN. The performance and validity of this framework are verified by real data sets of automobile main reducer. Experimental comparison results demonstrate that the diagnostic framework is more advantageous to conventional models.

The remaining content is organized into 4 extra sections. In Section 2, the fundamental theories of the proposed method are presented. The architecture of the proposed diagnostic framework is given in Section 3.  Section 4 gives the illustration of experimental dataset and provides comparative and detailed analysis of the diagnostic performance. In Section 5, the conclusions are presented.

## 2. The Fundamental Theories

### 2.1. Basic Autoencoder

**Figure 1:**
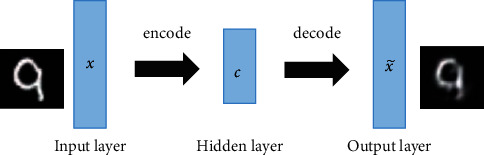
Structure of basic autoencoder.

Autoencoder is a typical unsupervised deep learning algorithm with asymmetrical neural network structure, and it is mainly utilized in deep feature extraction and dimension reduction [35–37]. The basic architecture of autoencoder contains three conjoint layers: an input layer, a hidden layer, and an output layer [38]. There are two stages in the unsupervised feature learning: encode and decode. As shown in Figure 1, the representative features *c* in lower dimensionality can be learned from unlabeled original input data *x* by encode. Then, in the stage of decode, a reconstruction of input data $\tilde{x}$ is generated from the learned feature *c*.

With sigmoid function, the original input data represented as *x*=[*x*_1_, *x*_2_,…,*x*_*n*_]^*T*^ are transformed into a new vector of representation *c*=[*c*_1_, *c*_2_,…,*c*_*p*_]^*T*^ as follows:

(1) $$\begin{array}{c} c=\text{sig}\left(Wx+b\right), \end{array}$$

where *W* is the weight matrix between the first layer and the second layer of autoencoder and *b* is the bias vector of neural network. Then, the reconstruction vector $\tilde{x}={\left[{\tilde{x}}_{1},{\tilde{x}}_{2},\ldots ,{\tilde{x}}_{n}\right]}^{T}$ is formatted from feature vector *c* as follows:

(2) $$\begin{array}{c} \tilde{x}=\text{sig}\left(\tilde{W}c+\tilde{b}\right), \end{array}$$

where $\tilde{W}$ is the weight matrix between the second layer and the third layer of autoencoder and $\tilde{b}$ is the bias vector of neural network. During the unsupervised process of feature extraction, parameter vector [*W*, *b*, $\tilde{W}$, $\;\tilde{b}$] is optimized by minimizing the training error between the original data *x* and the reconstruction data $\tilde{x}$.

### 2.2. Convolutional Autoencoder

**Figure 2:**
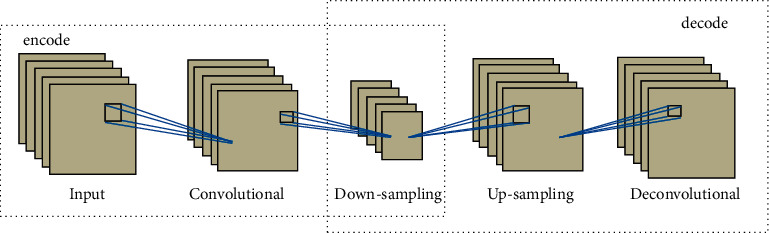
Structure of CAE.

As a special variant of basic autoencoder, convolutional autoencoder (CAE) is widely used in feature extraction of image and data in high dimensionality [39, 40]. As shown in Figure 2, without fully connected layers, CAE consists of input layer, convolutional layer, down-sampling layer, up-sampling layer, and deconvolutional layer. Through adding advantages of CNN into the unsupervised feature learning structure of autoencoder, CAE can extract the local characteristic structures and latent features from input data using a series of alternate convolutional layers and down-sampling layers for the encoding process [41, 42]. For input vector *X*, a convolutional layer with *K* convolutional filters maps *X* into *K* hidden feature maps. The *k*th feature map is represented as follows:

(3) $$\begin{array}{c} h_{k}=\sigma \left(X*W_{k}+b_{k}\right),\;k=1,2,\ldots K, \end{array}$$

where *W*_*k*_ and *b*_*k*_ are the *k*th kernel filter and bias and *σ*( ) represents nonlinear function. By employing *L* convolutional layers, the final latent deep features of *X* are extracted recursively and expressed as follows:

(4) $$\begin{array}{c} h^{L}=\sigma \left(h^{L-1}*W^{L}+b^{L}\right), \end{array}$$

where *h*^*L*−1^ is the hidden feature obtained from the (*L* − 1)th convolutional layer.

A series of up-sampling layers and deconvolutional layers are used to reconstruct raw input data from *h*^*L*^ in the process of decoding. The output of the last deconvolutional layer is as follows:

(5) $$\begin{array}{c} Y^{L}=\sigma \left(Y^{L-1}*W^{{\;}^{\prime }L}+b^{{\;}^{\prime }L}\right). \end{array}$$

CAE utilizes mean square error (MSE) [43] between original data *X* and reconstruction data *Y* as the loss function, and the convolutional filter and bias of convolutional layers are fine-tuning during the unsupervised feature learning. The loss function is optimized as follows:

(6) $$\begin{array}{c} L\left(W,b\right)=\underset{W,b}{\min}\frac{1}{2N}X-Y^{2}. \end{array}$$

## 3. Proposed Methodology

### 3.1. The Proposed Diagnostic Framework

**Figure 3:**
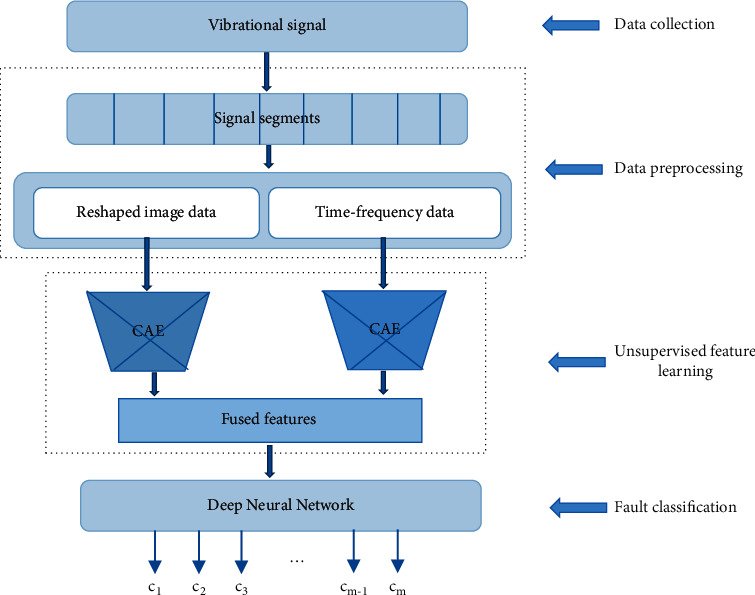
The proposed framework.

As shown in Figure 3, the proposed diagnostic framework contains four main phases: data collection, data preprocessing, unsupervised feature learning based on PCAE, and fault classification based on DNN. In the first phase, vibrational signals are collected from a test rig which simulates the real working conditions of main reducer. The valuable representative features for distinguishing normal condition and various failure conditions are deeply embedded in the raw signals.

### 3.2. Data Preprocessing

In intelligent fault diagnosis, the vibrational signal preprocessing contains three preparation steps: normalization, segmentation, and reshaping. After collecting the raw vibrational signals, data normalization should be implemented to restrain values within a limited range so as to reduce the fluctuation of training. A frequently used normalization approach is *z*-score normalization based on statistical properties of data, which utilizes the standard deviation *σ* and mean value  *μ* as follows:

(7) $$\begin{array}{c} \tilde{x}=\frac{x-\mu }{\sigma }. \end{array}$$

To enhance the size of dataset, the normalized data should be segmented into a series of fixed-size segments containing *N* points using sliding window. The segments with the length of *N* are used as sample set to train the unsupervised feature learning model.

In order to perform unsupervised feature learning based on convolutional autoencoder, one-dimensional sample set obtained from the process of segmentation needs to be reshaped into two-dimensional image form. The reshaping procedure can be represented as follows:

(8) $$\begin{array}{c} N—>\sqrt{N}\times \sqrt{N}. \end{array}$$

### 3.3. Unsupervised Feature Learning Based on PCAE

#### 3.3.1. Parallel Architecture for Unsupervised Feature Learning

**Figure 4:**
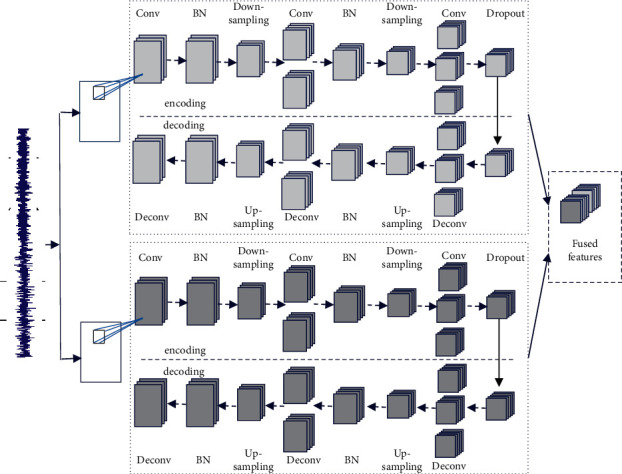
Architecture of the model based on PCAE.

In order to extract deep and representative features which are sensitive to various fault modes, this research constructs a parallel unsupervised feature learning model with two branches. One branch is used to directly perform feature extraction from segmented data in time domain, while in the other branch features are extracted from spectrograms in time-frequency domain which are obtained by using continuous wavelet transform. Two parallel branches based on CAE with different structures concurrently analyze the raw time-domain data to realize data compression and feature learning. Finally, the output features of two branches are fused together as the input of fault classifier. The architecture of the unsupervised feature learning model based on PCAE is shown in Figure 4.

As shown in Figure 4, the unsupervised feature learning model based on CAE contains a series of convolutional layers and down-sampling layers which are stacked alternately. For each convolutional layer, ReLU activation function is utilized to perform fast training and reduce the probability of gradient vanishing [43].

#### 3.3.2. Dropout Regularization

This research utilizes the dropout regularization technique in the training process of deep networks to avoid the occurrences of overfitting. As a typical stochastic regularization technology, some neural network units are temporarily discarded from the hidden layers according to a certain probability which is denoted as *ρ* in the range of (0, 1).

In the encoding stage, dropout layers are inserted after each pair of convolutional layer and down-sampling layer. Generally speaking, dropout is only used after the pooling layer. Based on the sparse weight vector, network complexity is decreased and generalization performance of testing set can be obviously enhanced.

#### 3.3.3. Batch Normalization

In deep learning, as the numbers of layers gradually increase, the shift of data distribution will lead to the phenomenon of internal covariate shift, which will affect the training speed [44]. Batch normalization is used in this research to handle this typical problem. The advantages of batch normalization in the training process of deep neural network are accelerating the training process, alleviating the dependences of initial parameters, preventing overfitting, and improving the network generalization.

In batch normalization layers which are placed after convolutional layers, features are divided into several groups to update the parameters according to the group. The data in one group together determine the direction of the gradient and reduce the randomness when descending. On the other hand, because the sample number of batches is much smaller than that of the whole data set, the computational cost is much lower.

### 3.4. Fault Classification Based on DNN

After the latent features are extracted from the unsupervised feature learning model based on PCAE, a fault classification model is construced based on deep neural networks (DNNs). DNN is a typical supervised learning model containing a series of fully connected hidden layers. With the purpose of feature compression and fault recognition, the size of hidden layers is on the decrease layer-by-layer. The network weights in each layer are randomly initialized and are further trained by using the fused feature vectors obtained from PCAE and their corresponding fault labels. Errors between real labels and network outputs are back propagated to adjust the network weights iteratively.

To improve the training efficiency, in this research, classification error rate is used as loss function as follows:

(9) $$\begin{array}{c} L=\frac{\sum_{i=1}^{N}\left(y_{i}==\hat{y_{i}}\right)}{N}, \end{array}$$

where *y*_*i*_ represents the target label and $\hat{y_{i}}$ is the network output. For complex network with multiple hidden layers, each layer has a large amount of weights. If the size of sample set is limited, overfitting is prone to occur. *L*2 regularization is an effective method to handle the problem of overfitting. By adding *L*2 regularization term, the cost function is as follows:

(10) $$\begin{array}{c} L=\frac{\sum_{i=1}^{N}\left(y_{i}==\hat{y_{i}}\right)}{\lambda \sum_{w}W^{2}}, \end{array}$$

where *λ* denotes the decay hyperparameter.

## 4. Experiments and Analysis

### 4.1. Experimental Environment and Dataset Preparation

**Figure 5:**
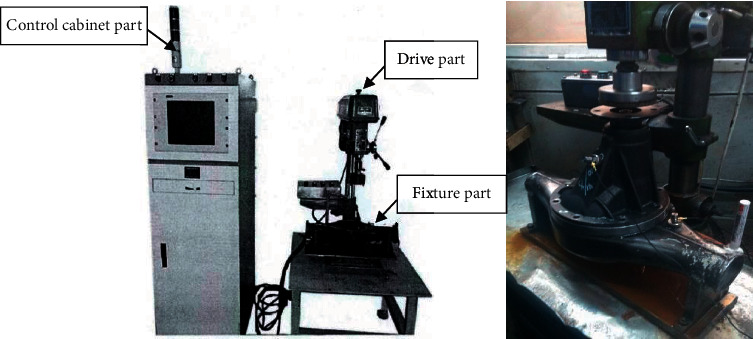
The test rig.

**Figure 6:**
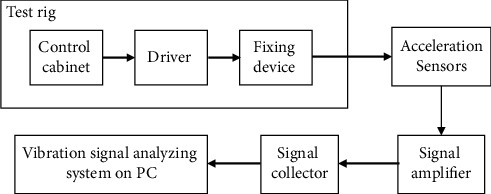
The schematic diagram of experimental setup.

The experiment analysis is implemented based on actual vibrational signals which are collected from a main reducer test rig. The test rig collects vibration signals of normal mode and various failure modes by simulating the practical working condition of the main reducer. As shown in Figure 5, there are three essential components in the test rig: a control cabinet which is used to control the running state and rotating speed, a driver which is used to drive the running of motor, and a fixing device which can fix the main reducer and simulate the actual installation of the main reducer so as to reflect its motion status. In this research, we adopt vibrational signals measured by two accelerometers (a vertical accelerometer and a horizontal accelerometer) as the dataset. The schematic diagram of experimental setup is shown in Figure 6. The collected vibration signal of main reducer is obtained by the acceleration sensors. To enhance the output signal of the sensor, the signal was amplified using a signal amplifier and then inputted into signal collector.

As shown in Table 1, according to previous research studies of main reducer for automobile rear axle, we select 6 failure modes which are the most frequently occurred for main reducer in actual case. Vibrational signals for 7 condition modes (6 failure modes and a health mode) are collected at the rotating speed of 800r/min. The sampling frequency *f*_*s*_ is set to 12 kHz. In order to reserve useful information of failure modes, the sampling frequency should not be less than the meshing frequency of gear.

By using the test rig to mimic the real running and operation of the faulty main reducer and the normal main reducer, we collect a series of vibration signals with the duration of 10 seconds. To ensure signal accuracy, it is better to start sampling with a delay of 0.5 seconds. Each sample corresponding to 7 condition modes contains 120000 data points. According to the rotation frequency and cycle of main reducer, each sample is segmented into 300 segmentations in which each segmentation contains 400 data points to cover a full cycle of rotation.

For each condition mode, 10 tests are performed repeatedly to collect enough data to represent the mode. Therefore, there are 3000 segmentations for each condition mode. During the preprocessing, the data size is reshaped from 400 to 20 × 20 which can be inputted into CAE to extract the latent features from original time-domain data.

The implementation of the experimental analysis is achieved in Matlab 8.0 on the PC with CPU of 3.4 GHz and RAM of 4 GB.

### 4.2. Architecture Design

The unsupervised feature learning model based on PCAE consists of two parallel CAE branches in which one CAE can automatically learn embedded features from reshaped images of original vibration signals in time domain and another CAE can automatically learn features from spectrogram of time-frequency domain. Each CAE contains convolutional layers, down-sampling layers or up-sampling layers, batch normalization layers, and dropout layers. In the architecture of CAE, the convolutional layers with ReLU activation function are utilized to extract feature maps, and the subsequent down-sampling layers are utilized to adjust the size of feature maps. The representative features extracted from two parallel branches of PCAE are fused together and inputted into the classifier based on DNN.

The structure and parameters of the proposed model based on PCAE and DNN are given in Table 2. In the parallel feature learning model based on PCAE, three convolutional layers with different sizes of convolution kernel are consecutively implemented to learn feature maps. Meanwhile, the quantity of convolution kernels is twice as much as the upper convolutional layer so as to make the feature maps smaller and thicker. Batch normalization and dropout with the rate of 0.5 are employed after convolutional operations to prevent overfitting.

In the fault classifier based on DNN, the fused features obtained from PCAE are flattened into one-dimensional and inputted into three full connection layers with randomly initial parameters. With a softmax layer, a predicted vector which can reflect the type of fault is finally outputted from the fault classifier.

During the training process, some hyperparameters of the proposed model are set into optimal fixed values based on previous research and comparative analysis. Hyperparameters of the proposed model are given in Table 3.

### 4.3. Experimental Results and Comparative Analysis

#### 4.3.1. Basic Analysis of the Diagnostic Performance

To analyze the diagnostic performance of the proposed model, a series of comparative experiments are implemented. The sample set of 7 condition modes is divided into two subsets. Most of the sample set is used to be the training set, and the rest is regarded as testing set. With the purpose of analyzing the diagnostic capability of the proposed model on the whole sample set, 5-fold cross validation is utilized to generate five different combinations of training set and testing set. For each combination, the proposed model is trained on 80% data which are randomly selected and tested on the other 20% data. Experimental results of five individual trials are given in Table 4.

It can be seen from the results in Table 4 that the proposed model can obtain excellent classification performance. The training accuracy of five trials is within the range of 97% to 99%, and the testing accuracy is within the range of 95% to 97%. Meanwhile, the classification capability is stable without obvious fluctuation. It can be proved that the proposed model can obtain sustained diagnostic performance even if the combinations of training set and testing set are various.

**Figure 7:**
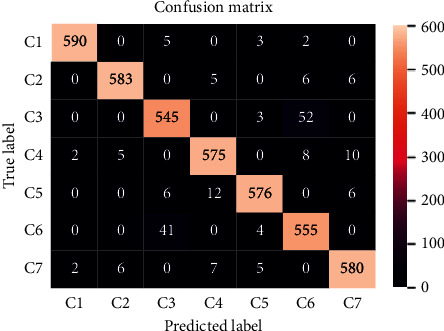
Confusion matrix of the diagnostic prediction.

To analyze the fault recognition capability of the proposed model for 7 different fault modes, a series of fault predictions on 7 fault modes of the testing set are implemented. The confusion matrix of the prediction results is given in Figure 7.

It can be observed that the vast majority of samples of C1 (health mode) are correctly predicted. It means that the differentiation degree of health mode against other fault modes is highest. Among the rest of 6 failure modes, C3 and C6 are more confusing and misclassified. Among 600 samples of C3, there are 52 samples which are predicted to C6. Overall, the proposed model can correctly recognize most of the faulty samples in testing set.

#### 4.3.2. Comparison with State-of-the-Art Models

To validate the superiority of the proposed model, a set of comparative experiments corresponding to several state-of-art models are carried out. In the field of condition monitoring and intelligent fault diagnosis, BPNN, SVM, and CNN are frequently used methods to build diagnostic models. For comparison, we construct diagnostic models based on BPNN, SVM, and CNN using raw time domain vibration signals. The proportion between training set and testing set is 4 to 1. Each model is tested for five trials. The average training accuracy and average testing accuracy of five trials are tallied as shown in Table 5.

It can be clearly concluded from Table 5 that the proposed model based on PCAE-DNN takes the best accuracy both on two subsets. The testing accuracy of PCAE-DNN is 95.86% which is nearly 6% higher than other three models. Among these comparative models, the average accuracy of the CNN-based model which is trained by training set is 93.26%, and the average accuracy of the CNN-based model which is tested by testing set is 90.52%. The two accuracies of the abovementioned CNN-based model are superior to the shallow model based on SVM and BPNN because of its deep structure of neural network.

It follows that deep structure of neural network has the natural advantage of automatic and adaptive feature learning. In addition, the proposed model combines two parallel branches to extract deep feature maps from time domain data and time-frequency domain data so that more comprehensive and pivotal features can be excavated. After that, the deep features are fed into another deep neural network with several fully connected layers to output the fault labels.

#### 4.3.3. Performance Analysis with Various Sizes of Dataset

For a diagnosis system, it is a significant advantage that using the limited training sample set can still obtain good diagnostic accuracy. Based on this, a series of comparative experiments with different datasets are carried out. Several models with the same parameters were built using 100%, 70%, 40%, 20%, and 5% of the original sample set, respectively. In the reduced dataset, 80% of the selected data are used to train the models and the rest data are used to test the models. Each situation is implemented for five times, and the average results of five trials were compared and analyzed as shown in Table 6.

It can be clearly displayed that for each situation, the precision of the proposed model based on PCAE-DNN is obviously better than that of other models. Meanwhile, when the dataset size is reduced, the accuracy of each model decreases more or less. With only 5% data, the diagnostic accuracy of the model based on BPNN is decreased from 89.54% to 61.91% and the diagnostic accuracy of the model based on SVM is decreased from 90.15 to 63.72%. The results demonstrate that the difference between models based on PCAE-DNN with 5% data and 100% data is relatively small, while the difference between models based on CNN, BPNN, and SVM which are built with 5% data and 100% data is huge. It is particularly noticeable that the proposed model based on PCAE-DNN can maintain good performance even with minimal dataset. The main reason for the superiority is that the proposed model is built with deep features which are fused from time-domain data and time-frequency domain data.

**Figure 8:**
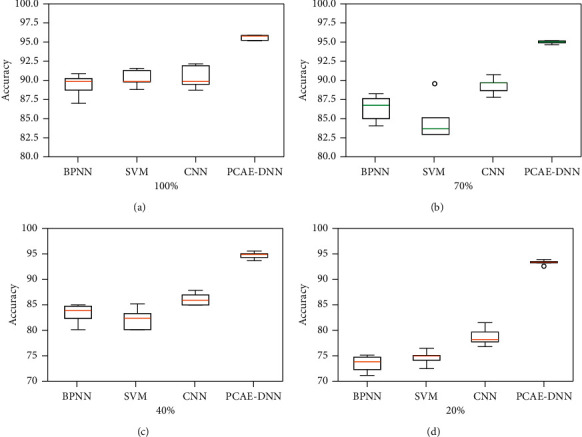
Performance of models with various dataset sizes: (a) 100% dataset; (b) 70% dataset; (c) 40% dataset; (d) 20% dataset.

With the purpose of analyzing the stability of these models with various sizes of training set, we plot the box-plots of diagnostic accuracy as illustrated in Figure 8. It can be seen that for five trials, the model based on PCAE-DNN can obtain great results in all cases. For 100% dataset case, the variance of the model based on CNN is much higher than other models, while for 70% dataset case, the variance of the model based on BPNN significantly increased. Overall, with the limited training sample set, the proposed model can still obtain consistent diagnostic accuracy.

#### 4.3.4. Effectiveness of the Unsupervised Feature Learning

A key point of the proposed model is that the deep feature maps are learned from time domain data and time-frequency domain data in unsupervised way. Feature maps extracted from two parallel branches based on CAE are fused together and inputted into a structure based on DNN to achieve fault recognition. In order to measure the effectiveness of the unsupervised feature learning scheme, a series of experiments are performed to compare the capability of the proposed parallel branch feature extraction and single branch feature extraction.

The proposed model is based on PCAE-CWT in which one branch employs CWT to obtain spectrograms of time-frequency domain as early manual feature extraction. To prove the superiority of CWT, an alternative parallel model based on PCAE-EMD is constructed. In addition, two single branch models based on CAE-CWT and CAE-signal are used to complete comparison and analysis. For each model, five trials are implemented, and the average accuracy is given in Table 7.

According to the results in Table 7, the performance of the CAE-based model with raw vibration signals as input is the worst. By adding the manual feature extraction method CWT into the CAE-based model, the average testing accuracy of the model based on CAE-CWT increases from 90.41% to 91.69%. A conclusion can be drawn that to some extent CWT extracts certain features which are beneficial to fault classification. The model based on PCAE-EMD shows measurable improvement in accuracy on both training set and testing set which is 95.23% and 93.05%. Nevertheless, the performance of PCAE-EMD is still inferior to the proposed model, that is 95.86%. The comparative results highlight the effectiveness of parallel feature fusion and the significance of early feature extraction using CWT.

**Figure 9:**
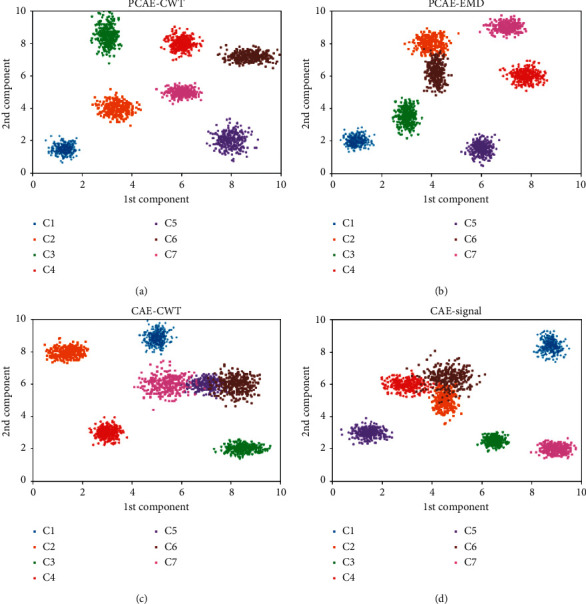
Dispersion degree comparisons of principal features.

With the purpose of visualizing the dispersion of the deep features extracted from each model more intuitively and clearly, we utilize principal component analysis (PCA) to reduce dimension of the features learned from basic CAE and retain two principal components. Then, these principal components are projected into two-dimensional feature space. The dispersion degree comparisons of principal features are shown in Figure 9.

As shown in Figure 9(a), two principle components of the features learned from PCAE-CWT show the obvious characteristics of intraclass aggregation and interclass separation. It means that such features can achieve optimal classification accuracy as input of fault classifier. As shown in Figure 9(b), there is a little of overlap between principal features of C2 and C6. It can be concluded that the features learned from PCAE-EMD are slightly inferior to the proposed model using CWT and parallel branches of feature learning. As shown in Figure 9(c), features are overlapped between two adjacent classes. As shown in Figure 9(d), features of C2, C4, and C6 are overlapped. It means that for models with single branch of feature learning, features show obvious less aggregation within classes and separation between classes than PCAE-CWT. The comparison results reflect the excellent performance of the proposed unsupervised feature learning method.

## 5. Conclusions

In this research, we propose a novel unsupervised feature learning model fault based on parallel convolutional autoencoder (PCAE) and by combining deep neural network (DNN) with PCAE to recognize fault. Firstly, deep features are, respectively, extracted from reshaped form of raw sample set in time domain and from spectrogram in time-frequency domain by two parallel unsupervised feature learning branches based on convolutional autoencoder (CAE). Furthermore, during the training process of PCAE, dropout regularization and batch normalization are utilized to prevent over fitting. Finally, extracted representative features are fed into the classification model based on DNN with softmax. The proposed model based on PCAE-DNN can learn more salient deep features from data. The results of comparison experiments show that the fault classification accuracy of the proposed model is better than that of other state-of-art models. Even with small data sets, the model based on PCAE-DNN can achieve better performance and stability. In the future, works on developing simultaneous fault diagnosis framework based on deep learning models and exploring new ways of converting vibrational signals into images will be carried out.

## Figures and Tables

**Table 1:** Operation condition modes.

Labels	Condition modes
C1	Health mode
C2	Normal status
C3	Gear error
C4	Gear burr
C5	Gear hard point
C6	Misalignment
C7	Gear tooth broken

**Table 2:** The architecture and parameter values of the proposed model.

Model	Original vibrational signals			
	Reshaped images in time domain		Spectrogram in time-frequency domain	
Parallel feature learning model based on PCAE	Conv1	16 × 12 × 12	Conv1	16 × 12 × 12
	BN1	—	BN1	—
	Down-samp1	Pool size = 2 × 2	Down-samp1	Pool size = 2 × 2
	Dropout1	0.5	Drop1	0.5
	Conv2	32 × 6 × 6	Conv2	32 × 6 × 6
	BN2	—	BN2	—
	Down-samp2	Pool size = 2 × 2	Down-samp2	Pool size = 2 × 2
	Conv3	64 × 6 × 6	Conv3	64 × 6 × 6
	Dropout2	0.5	Drop2	0.5

Fault classifier based on DNN	Flatten layer		—	
	Full connection layer 1		32	
	Full connection layer 2		16	
	Full connection layer 3		7	
	Softmax		N/A	
	Output		Predicted vector	

**Table 3:** Hyperparameters of the proposed model.

Hyperparameters	Values
Learning rate	0.001
Pool size	2 × 2
Epochs of pretraining	30
Epochs of fine-tuning	60

**Table 4:** Diagnosis accuracy of the proposed model.

Trials	Diagnostic performance	
	Training accuracy (%)	Testing accuracy (%)
1	97.43	95.17
2	98.28	95.85
3	98.81	96.29
4	97.67	95.06
5	98.59	96.38

**Table 5:** Fault diagnostic performance of different models.

Models	Training accuracy (%)	Testing accuracy (%)
BPNN	91.79	89.54
SVM	92.52	90.15
CNN	93.26	90.52
PCAE-DNN	98.18	95.86

**Table 6:** Precision of different situations.

Models	BPNN	SVM	CNN	PCAE-DNN
Dataset size	Average testing accuracy (%)			
100%	89.54	90.15	90.52	95.86
70%	86.39	85.66	88.83	95.04
40%	83.22	82.83	84.37	94.39
20%	73.07	74.47	78.69	93.12
5%	61.91	63.72	73.15	92.47

**Table 7:** Performance of various models.

Models	Training accuracy (%)	Testing accuracy (%)
PCAE-CWT	98.18	95.86
PCAE-EMD	95.23	93.05
CAE-CWT	92.47	91.69
CAE-signal	91.83	90.41

## Data Availability

The data used to support the findings of this study are available from the corresponding author upon request.

## References

[1] Yao L.-J., Ding J.-X. (2007). An on-line vibration monitoring system for final drive of automobile. *Noise and Vibration Control*.

[2] Ye Q., Pan H., Liu C. (2015). A framework for final drive simultaneous failure diagnosis based on fuzzy entropy and sparse Bayesian extreme learning machine. *Computational Intelligence and Neuroscience*.

[3] Ye Q., Liu C. (2020). A multichannel data fusion method based on multiple deep belief networks for intelligent fault diagnosis of main reducer. *Symmetry*.

[4] Lee J., Wu F. J., Zhao W., Ghaffari M., Liao L. X., Siegel D. (2014). Prognostics and health management design for rotary machinery systems: reviews, methodology and applications. *Mechanical Systems and Signal Processing*.

[5] Tahan M., Tsoutsanis E., Muhammad M., Abdul Karim Z. A. (2017). Performance-based health monitoring, diagnostics and prognostics for condition-based maintenance of gas turbines: a review. *Applied Energy*.

[6] Sen A., Majumder M. C., Mukhopadhyay S., Biswas R. K. (2017). Condition monitoring of rotating equipment considering the cause and effects of vibration: a brief review. *International Journal of Modern Engineering and Research Technology*.

[7] Attoui I., Oudjani B., Boutasseta N., Fergani N., Bouraiou A. (2020). Novel predictive features using a wrapper model for rolling bearing fault diagnosis based on vibration signal analysis. *International Journal of Advanced Manufacturing Technology*.

[8] Grasso M., Chatterton S., Pennacchi P., Colosimo B. M. (2016). A data-driven method to enhance vibration signal decomposition for rolling bearing fault analysis. *Mechanical Systems and Signal Processing*.

[9] Sousa R., Antunes J., Coutinho F., Silva E., Santos J., Ferreira H. (2019). Robust cepstral-based features for anomaly detection in ball bearings. *International Journal of Advanced Manufacturing Technology*.

[10] Singh J., Darpe A. K., Singh S. P. (2018). Rolling element bearing fault diagnosis based on over-complete rational dilation wavelet transform and auto-correlation of analytic energy operator. *Mechanical Systems and Signal Processing*.

[11] Moumene I., Ouelaa N. (2016). Application of the wavelets multiresolution analysis and the high-frequency resonance technique for gears and bearings faults diagnosis. *International Journal of Advanced Manufacturing Technology*.

[12] Yeap Y. M., Ukil A. Fault detection in HVDC system using short time fourier transform.

[13] Islam M. M. M., Kim J.-M. (2019). Automated bearing fault diagnosis scheme using 2D representation of wavelet packet transform and deep convolutional neural network. *Computers in Industry*.

[14] Liang P., Deng C., Wu J. (2019). Compound fault diagnosis of gearboxes via multi-label convolutional neural network and wavelet transform. *Computers in Industry*.

[15] Sun J., Yan C., Wen J. (2018). Intelligent bearing fault diagnosis method combining compressed data acquisition and deep learning. *IEEE Transactions on Instrumentation and Measurement*.

[16] Chen X., Wang S., Qiao B., Chen Q. (2018). Basic research on machinery fault diagnostics: past, present, and future trends. *Frontiers of Mechanical Engineering*.

[17] Liu R., Yang B., Zio E., Chen X. (2018). Artificial intelligence for fault diagnosis of rotating machinery: a review. *Mechanical Systems and Signal Processing*.

[18] Zhu K., Song X., Xue D. (2014). A roller bearing fault diagnosis method based on hierarchical entropy and support vector machine with particle swarm optimization algorithm. *Measurement*.

[19] Jia F., Lei Y., Guo L., Lin J., Xing S. (2018). A neural network constructed by deep learning technique and its application to intelligent fault diagnosis of machines. *Neurocomputing*.

[20] Qi Y., Shen C., Wang D., Shi J., Jiang X., Zhu Z. (2017). Stacked sparse autoencoder-based deep network for fault diagnosis of rotating machinery. *IEEE Access*.

[21] Liu G., Bao H., Han B. (2018). A stacked autoencoder-based deep neural network for achieving gearbox fault diagnosis. *Mathematical Problems in Engineering*.

[22] Hoang D.-T., Kang H.-J. (2019). Rolling element bearing fault diagnosis using convolutional neural network and vibration image. *Cognitive Systems Research*.

[23] Abdeljaber O., Avci O., Kiranyaz S., Gabbouj M., Inman D. J. (2017). Real-time vibration-based structural damage detection using one-dimensional convolutional neural networks. *Journal of Sound and Vibration*.

[24] Xia M., Li T., Shu T., Wan J., De Silva C. W., Wang Z. (2019). A two-stage approach for the remaining useful life prediction of bearings using deep neural networks. *IEEE Transactions on Industrial Informatics*.

[25] Al-Dulaimi A., Zabihi S., Asif A., Mohammadi A. (2019). A multimodal and hybrid deep neural network model for Remaining Useful Life estimation. *Computers in Industry*.

[26] Zhao R., Yan R., Chen Z., Mao K., Wang P., Gao R. X. (2019). Deep learning and its applications to machine health monitoring. *Mechanical Systems and Signal Processing*.

[27] Shao H., Jiang H., Zhao H., Wang F. (2017). A novel deep autoencoder feature learning method for rotating machinery fault diagnosis. *Mechanical Systems and Signal Processing*.

[28] Godói L. F. d., Nóbrega E. G. d. O. (2021). Denoising convolutional autoencoder configuration for condition monitoring of rotating machines. *Journal of the Brazilian Society of Mechanical Sciences and Engineering*.

[29] Chen H., Wang Y.-H., Fan C.-H. (2021). A convolutional autoencoder-based approach with batch normalization for energy disaggregation. *The Journal of Supercomputing*.

[30] Aljemely A. H., Xuan J., Farqad K., Jawad J. (2020). A novel unsupervised learning method for intelligent fault diagnosis of rolling element bearings based on deep functional auto-encoder. *Journal of Mechanical Science and Technology*.

[31] Hinton G. E., Osindero S., Teh Y.-W. (2006). A fast learning algorithm for deep belief nets. *Neural Computation*.

[32] Peng K., Jiao R., Dong J., Pi Y. (2019). A deep belief network based health indicator construction and remaining useful life prediction using improved particle filter. *Neurocomputing*.

[33] DEUTSCH J., HE D. Using deep learning based approaches for bearing remaining useful life prediction.

[34] HU C.-H., PEI H., SI X.-S., Du D.-B., Pang Z.-N., Wang X. (2020). A prognostic model based on DBN and diffusion process for degrading bearing. *IEEE Transactions on Industrial Electronics*.

[35] Yu W., Kim I. Y., Mechefske C. (2019). Remaining useful life estimation using a bidirectional recurrent neural network based autoencoder scheme. *Mechanical Systems and Signal Processing*.

[36] Song Y., Xia T., Zheng Yu (2019). Remaining useful life prediction of turbofan engine based on autoencoder-BLSTM. *Computer Integrated Manufacturing Systems*.

[37] Wang Y., Yao H., Zhao S. (2016). Auto-encoder based dimensionality reduction. *Neurocomputing*.

[38] Shuaixin T. (2020). An intrusion detection method based on stacked autoencoder and support vector machine. *Journal of Physics Conference Series*.

[39] Yu Y, Long J., Cai Z. (2017). Network intrusion detection through stacking dilated convolutional autoencoders. *Security and Communication Networks*.

[40] Masci J., Meier U., Cireşan D., Schmidhuber J. Stacked convolutional auto-encoders for hierarchical feature extraction.

[41] Chen S., Yu J., Wang S. (2020). One-dimensional convolutional auto-encoder-based feature learning for fault diagnosis of multivariate processes. *Journal of Process Control*.

[42] Kim C., Park J. (2019). Designing online network intrusion detection using deep auto-encoder q-learning. *Computers & Electrical Engineering*.

[43] Rumelhart D. E., Hinton G. E., Williams R. J. (1988). Learning representations by back-propagating errors. *Neurocomputing: Foundations of Research*.

[44] Serwa A. (2017). Studying the effect of activation function on classification accuracy using deep artificial neural networks. *Journal of Remote Sensing & GIS*.

